# Efficient genomics*-*based ‘end*-*to*-*end’ selective tree breeding framework

**DOI:** 10.1038/s41437-023-00667-w

**Published:** 2024-01-03

**Authors:** Yousry A. El-Kassaby, Eduardo P. Cappa, Charles Chen, Blaise Ratcliffe, Ilga M. Porth

**Affiliations:** 1 Faculty of Forestry, The University of British Columbia, Vancouver, BC Canada; 2 Instituto Nacional de Tecnología Agropecuaria (INTA), Instituto de Recursos Biológicos, Centro de Investigación en Recursos Naturales, Buenos Aires, Argentina; 3 Consejo Nacional de Investigaciones Científicas y Técnicas (CONICET), Buenos Aires, Argentina; 4 Department of Biochemistry and Molecular Biology, Oklahoma State University, Oklahoma, OK USA; 5 Department of Wood and Forest Sciences, Université Laval, Quebec, QC Canada

**Keywords:** Genetics, Plant breeding

## Abstract

Since their initiation in the 1950s, worldwide selective tree breeding programs followed the recurrent selection scheme of repeated cycles of selection, breeding (mating), and testing phases and essentially remained unchanged to accelerate this process or address environmental contingencies and concerns. Here, we introduce an “end-to-end” selective tree breeding framework that: (1) leverages strategically preselected GWAS-based sequence data capturing trait architecture information, (2) generates unprecedented resolution of genealogical relationships among tested individuals, and (3) leads to the elimination of the breeding phase through the utilization of readily available wind-pollinated (OP) families. Individuals’ breeding values generated from multi-trait multi-site analysis were also used in an optimum contribution selection protocol to effectively manage genetic gain/co-ancestry trade-offs and traits’ correlated response to selection. The proof-of-concept study involved a 40-year-old spruce OP testing population growing on three sites in British Columbia, Canada, clearly demonstrating our method’s superiority in capturing most of the available genetic gains in a substantially reduced timeline relative to the traditional approach. The proposed framework is expected to increase the efficiency of existing selective breeding programs, accelerate the start of new programs for ecologically and environmentally important tree species, and address climate-change caused biotic and abiotic stress concerns more effectively.

## Introduction

Forest tree selective breeding programs follow the recurrent selection scheme, involving repeated cycles of selection, breeding, and testing (Allard 1960; White et al. 2007) along with a product development phase for improved seed production (El-Kassaby 1995). While this process has successfully delivered substantial gains worldwide, it is highly structured and long-term, thus less responsive to addressing the pressing environmental contingencies (Wheeler et al. 2015; Matallana-Ramirez et al. 2021). In particular, climate change-induced biotic and abiotic stresses (Surówka et al. 2020) with their cascading biological consequences affecting populations’ survival and recruitment. These challenges require faster genetic evaluation methods that traditional selective breeding cannot provide. Thus, developing agile evaluation methods to address these new challenges necessitates efficient approaches that leverage advanced genomic capabilities and their integration into traditional selective breeding programs (Grattapaglia et al. 2018). Most tree selective breeding programs are protracted as they require creating structured pedigree (half- (HS) and full-sib (FS) families) during the breeding phase and necessitate long-term evaluation phases as most sought-after target traits (e.g., volume and wood density) are expressed at an advanced age (White et al. 2014). However, certain innovations have been effective in shortening the breeding cycle length to some extent. These include: (i) reliance on juvenile-mature correlations where early age performance serves as proxy to advanced age (Lambeth 1980), (ii) utilizing open-pollinated (OP) families to bypass the structured pedigree requirement (Stonecypher et al. 1964), and (iii) applying pedigree reconstruction to assemble a “structured pedigree” from naturally produced offspring (a.k.a., “Breeding without Breeding”), thereby eliminating the breeding phase (El-Kassaby and Lstibůrek 2009; also see Grattapaglia et al. 2004).

Pedigree-dependent quantitative genetics analyses utilize the average numerator relationship (***A***-matrix), reflecting the contemporary genealogical relationships among the structured pedigree members (Wright 1922). This matrix is then used to estimate the genetic variance components using Restricted Maximum Likelihood (Gilmour et al. 1995) for predicting individuals’ breeding value using the Best Linear Unbiased Prediction algorithms (BLUP) (Henderson 1975). For monoecious species, when OP families are used, a half-sib family structure is assumed, with members of each OP family sired by different males, an inconceivable assumption considering trees’ pollination biology and ecology. In reality, OP families’ offspring often represent a mixture of self-sibs (progeny from self-pollination), half-sibs, and full-sibs with varying proportions, resulting in inflated additive genetic variance and heritability estimates (Namkoong 1966; Squillace 1971; Askew and El-Kassaby 1994). Another drawback of the average numerator relationship is its inability to differentiate among siblings within HS or FS families as it applies global relationship estimates without considering the Mendelian sampling term, which represents relatedness variation among FS and HS family members that cannot be determined by traditional pedigree analyses (Avendaño et al. 2005).

The availability of DNA sequence data has facilitated the accurate determination of the actual fraction of alleles shared between individuals (identity-by-state), enabling the estimation of their realized genomic pairwise kinship (***G***-matrix) (VanRaden 2008; also see Ritland 1996). The strength of the ***G***-matrix lies in its ability to capture genetic information from both contemporary and ancestral pedigrees, accurately accounting for hidden relationships that cannot be ascertained through the traditional contemporary pedigree analysis (Powell et al. 2010). This understanding has enabled the integration of DNA sequences into quantitative genetics where the pedigree-based relationship (***A***-matrix) is replaced by the genomic-based relationship (***G***-matrix). It is important to note that the ***G***-matrix application in OP families has successfully addressed the aforementioned drawbacks, resulting in accurate estimates of additive, dominance, and epistatic genetic variances (Gamal El-Dien et al. 2016).

Complex traits, following Fisher’s infinitesimal model, are often theorized to be controlled by a large number of genes, each explaining a small fraction of the trait’s variance (Fisher 1918). To harness the linkage disequilibrium (LD) between the traits’ causal genes and the genotypic data (genome-wide SNPs), genome-wide association studies (GWAS) have been used to unravel complex traits architectures and identify their underpinning causal genes (Visscher and Goddard 2019). However, the GWAS approach is statistically burdened by the multiple testing threshold, leading to failure in detecting many potentially causal genes with smaller effects as they do not meet the predetermined significance threshold (Tam et al. 2019).

Here, we present the genomic version of the “Breeding without Breeding” conceptualized by El-Kassaby and Lstibůrek (2009). This version represents a unified and all-encompassing selective breeding framework in a holistic “end-to-end” process that: (1) quantifies all the advantages of combining genomic data with the simple OP family structure, (2) generates reliable genetic information in a substantially reduced timeframe, (3) includes genetic evaluation and ranking, and (4) culminates in selecting the best individuals for further utilization. To illustrate this, we utilized phenotypic (tree height, diameter, and wood density) and genotypic (≈9 K SNPs) data from a 40-year-old spruce OP testing population growing on three sites in British Columbia, Canada (Gamal El-Dien et al. 2016). We then compared the results to those obtained using the traditional selective breeding scheme (i.e., ***A***-matrix).

## Materials and methods

### Genetic material, evaluated traits, DNA fingerprinting

A total of 1126, 40-year-old “Interior spruce” (*Picea glauca* (Moench) Voss × *P. engelmannii* Parry ex Engelm.) trees representing 25 open-pollinated (OP) families growing on three sites in Interior British Columbia, Canada (Aleza Lake: lat. 54° 03′ 15.7″ N, long. 122° 06′ 35.4″ W, elev. 700 m asl; Prince George Tree Improvement Station: lat. 53° 46′ 17.9″ N, long. 122° 43′ 07.6″ W, elev. 610 m asl; and Quesnel: lat. 52° 59′ 27.2″ N, long. 122° 12′ 30.6″ W, elev. 915 m asl). The field trial was established by the British Columbia Ministry of Forests, Forest Improvement and Research Management Branch, following a complete randomized block design with five to ten blocks and ten or fifteen tree-row-plots planted at 2.5 × 2.5 m spacing with a total of 181 OP families.

**Fig. 1:**
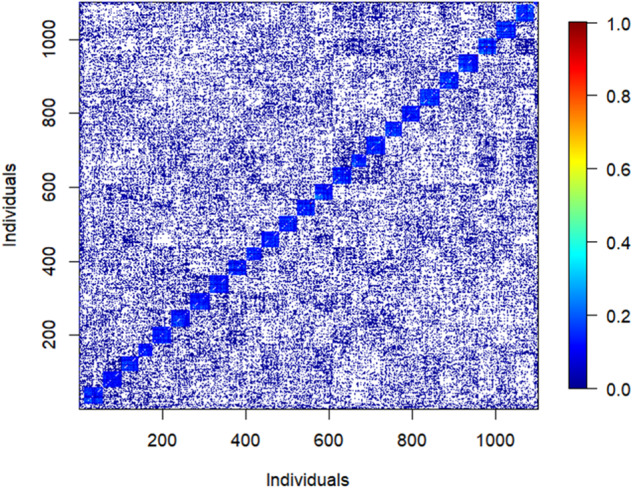
Heat map of pair*-*wise genomic relationship coefficients among 1101 individuals grouped by families. The heatmap demonstrates lack of genetic structure as families are presented by squares (blue) across the diagonal elements with off-diagonal representing shared ancestral pedigree among individuals.

The OP family testing method is the mass selection of individuals based on their desired phenotypic attributes, without prior knowledge of either their performance or pedigree. This selection method is conducted within a wide geographic area known as “breeding zone,” thus necessitating genetic testing to determine individuals’ genetic superiority (White et al. 2007). Offspring within OP families share a common parent (the seed donor) and are pollinated by the surrounding “local” pollen pool (male donors), often resulting in some detectable paternal relationships. Typically, OP family selection lacks a well-defined population structure, as only a limited number of individuals (seed donors) are selected from a specific location, as illustrated in Fig. 1 that depicts family structure and demonstrates the ancestral relationships among the entire test population.

It is essential to recognize that pedigree errors are often found in most breeding program (Adams et al. 1988; Devey et al. 2002; Doerksen and Herbinger 2008). Therefore, we leveraged the available genomic data to verify the pedigree in our genetic materials. This verification process led to several outcomes: (1) removal of 25 individual with low diagonal elements of the ***G***-matrix, (2) reassignment of 15 to different pedigree families as their genomic relatedness was found to be low, (3) creation of a new OP family consisting of 9 individuals, and (4) the identification of 13 individuals who did not belong to any of the initially studied 25 OP families (see genomic network below). As a result, the initial count of OP families of 25 increased by one, while the total number of individuals was reduced from 1126 to 1101. Pedigree errors are a commonly occurring issue during development and establishment of progeny testing materials that include processes such as seed-cone collection, seed extraction, seedling production, and progeny tests planting. Additionally, as the selected 25 families represent a subset of the initial 181 OP family test, it is reasonable to expect that the detected pedigree errors are part of the overall test.

From each site, four replications representing each of the originally studied 25 OP family were sampled and measured for: (1) total tree height (HT, in meters), (2) diameter at breast height (DBH, in centimeters), and (3) wood density (WD, g·cm^−3^) determined using X-ray densitometry (WD) from 5-mm bark-to-bark wood cores extracted at breast height in the north-south direction of each tree by increment borers (El-Kassaby et al. 2011). DNA extraction and Genotyping-by-Sequencing (GBS) (Elshire et al. 2011), details are available elsewhere (Gamal El-Dien et al. 2018). Here, we utilized a subset of the GBS-generated SNP data from the original file with 30% missing data (Ratcliffe et al. 2015) and selected those SNPs with the least missing data, then implemented mean imputation using the ‘A.mat’ function in the ‘rrBLUP’ R package (Endelman, 2011), resulting in a total of 8767 SNPs for quantitative genetic analyses (10.5061/dryad.7h44j101d).

### Quantitative genetics/genomics analyses

For computational efficiency, the statistical analyses were conducted in two stages. First, each trait was analyzed separately in each site using a pedigree-based classical a priori design model, where replications were fitted as a random effect. In the second stage, the phenotypic data adjusted for the design effects were obtained for each tree and trait and at each site by subtracting the estimated replication effects from the raw phenotype. Thereafter, the additive average-numerator (ABLUP) and genomic (GBLUP-A) best linear unbiased prediction analyses were performed for each of the three traits using the following additive multi-site individual-tree mixed model:

1 $$\left[\begin{array}{c}{{\boldsymbol{y}}}_{1}\\ {{\boldsymbol{y}}}_{2}\\ {{\boldsymbol{y}}}_{3}\end{array}\right]=\left[\begin{array}{ccc}{{\boldsymbol{X}}}_{1} & {\bf{0}} & {\bf{0}}\\ {\bf{0}} & {{\boldsymbol{X}}}_{2} & {\bf{0}}\\ {\bf{0}} & {\bf{0}} & {{\boldsymbol{X}}}_{3}\end{array}\right]\left[\begin{array}{c}{{\boldsymbol{\beta }}}_{1}\\ {{\boldsymbol{\beta }}}_{2}\\ {{\boldsymbol{\beta }}}_{3}\end{array}\right]+\left[\begin{array}{ccc}{{\boldsymbol{Z}}}_{{a}_{1}} & {\bf{0}} & {\bf{0}}\\ {\bf{0}} & {{\boldsymbol{Z}}}_{{a}_{2}} & {\bf{0}}\\ {\bf{0}} & {\bf{0}} & {{\boldsymbol{Z}}}_{{a}_{3}}\end{array}\right]\left[\begin{array}{c}{{\boldsymbol{a}}}_{1}\\ {{\boldsymbol{a}}}_{2}\\ {{\boldsymbol{a}}}_{3}\end{array}\right]+\left[\begin{array}{c}{{\boldsymbol{e}}}_{1}\\ {{\boldsymbol{e}}}_{2}\\ {{\boldsymbol{e}}}_{3}\end{array}\right]$$

where ${\boldsymbol{y}}{\boldsymbol{=}}\left[{{\boldsymbol{y}}}_{1}^{{\prime} }{\boldsymbol{,}}{{\boldsymbol{y}}}_{2}^{{\prime} }{\boldsymbol{,}}{{\boldsymbol{y}}}_{3}^{{\prime} }\right]$ is the vector of individual tree adjusted-phenotypes for the three sites; ${\boldsymbol{\beta }}=[{{\boldsymbol{\beta }}}_{1}^{{\prime} },{{{\boldsymbol{\beta }}}_{2}^{{\prime} }{\boldsymbol{,}}{\boldsymbol{\beta }}}_{3}^{{\prime} }]$ is the vector of site fixed effects (i.e., overall mean for each site); the additive genetic effects random vector of ${\boldsymbol{a}}=[{{\boldsymbol{a}}}_{1}^{{\prime} },{{{\boldsymbol{a}}}_{2}^{{\prime} }{\boldsymbol{,}}{\boldsymbol{a}}}_{3}^{{\prime} }]$ is distributed as ${\boldsymbol{a}}{\boldsymbol{ \sim }}{\boldsymbol{N}}\left({\boldsymbol{0}}{\boldsymbol{,}}{{\boldsymbol{\Sigma }}}_{{\boldsymbol{a}}}{\boldsymbol{\bigotimes }}{\boldsymbol{A}}\right)$, where ${{\boldsymbol{\Sigma }}}_{{\boldsymbol{a}}}$ is the genetic effects (co)variance matrix and ${\boldsymbol{A}}$ is the additive average-numerator relationship matrix containing the additive relationships among all trees (26 mothers without records plus 1101 offspring). Finally, ${\boldsymbol{e}}=[{{\boldsymbol{e}}}_{1}^{{\prime} },{{\boldsymbol{e}}}_{2}^{{\prime} },{{\boldsymbol{e}}}_{3}^{{\prime} }]$ is the vector of random residuals distributed as ${\boldsymbol{e}}{\boldsymbol{ \sim }}{\boldsymbol{N}}{\boldsymbol{(}}{\boldsymbol{0}}{\boldsymbol{,}}{{\boldsymbol{R}}}_{{\boldsymbol{0}}}{\boldsymbol{\bigotimes }}{\boldsymbol{I}}{\boldsymbol{)}}$ where ***R***_**0**_ is the residual (co)variance matrix for the three sites with dimension 3 × 3. We assumed an unstructured (co)variance matrix for the genetic effects (${{\boldsymbol{\Sigma }}}_{{\boldsymbol{a}}}$). The column vector of ***1***s ***X***_1_, ***X***_2_ and ***X***_3_, and the matrices ${{\boldsymbol{Z}}}_{{a}_{1}}$, ${{\boldsymbol{Z}}}_{{a}_{2}}$ and ${{\boldsymbol{Z}}}_{{a}_{3}}$ relate the observation to the means of the site effects in **β**, and the additive genetic effects for each tree in ***a***. The symbol (´) indicates the transpose operation.

The additive multi-trait multi-site individual-tree ABLUP-A model and the GBLUP models, which used a ***G***-matrix calculated using all available SNPs (8767; GBLUP-ALL) and 5628 SNPs selected by GWAS (based on their SNP absolute effect without imposing any variance contribution limits) (GBLUP-GWAS) (see below), respectively, were fitted as:

2 $$\left[\begin{array}{c}{{\boldsymbol{y}}}_{11}\\ \vdots \\ {{\boldsymbol{y}}}_{{ij}}\end{array}\right]=\left[\begin{array}{ccc}{{\boldsymbol{X}}}_{11} & {\bf{0}} & {\bf{0}}\\ {\bf{0}} & \ddots & {\bf{0}}\\ {\bf{0}} & {\bf{0}} & {{\boldsymbol{X}}}_{{ij}}\end{array}\right]\left[\begin{array}{c}{{\boldsymbol{\beta }}}_{11}\\ \vdots \\ {{\boldsymbol{\beta }}}_{{ij}}\end{array}\right]+\left[\begin{array}{ccc}{{\boldsymbol{Z}}}_{{a}_{11}} & {\bf{0}} & {\bf{0}}\\ {\bf{0}} & \ddots & {\bf{0}}\\ {\bf{0}} & {\bf{0}} & {{\boldsymbol{Z}}}_{{a}_{{ij}}}\end{array}\right]\left[\begin{array}{c}{{\boldsymbol{a}}}_{11}\\ \vdots \\ {{\boldsymbol{a}}}_{{ij}}\end{array}\right]+\left[\begin{array}{c}{{\boldsymbol{e}}}_{11}\\ \vdots \\ {{\boldsymbol{e}}}_{{ij}}\end{array}\right]$$

where ${\boldsymbol{y}}{\boldsymbol{=}}[{{\boldsymbol{y}}}_{11}^{{\prime} }{\boldsymbol{,}}{\boldsymbol{\cdots }}{\boldsymbol{,}}{{\boldsymbol{y}}}_{{ij}}^{{\prime} }]$ is the vector of adjusted-phenotypes for each *i* trait (*i* = DBH, HT and WD) and *j* site (*j* = 1, 2, 3); ${\boldsymbol{\beta }}=[{{\boldsymbol{\beta }}}_{11}^{{\prime} },{{\boldsymbol{\cdots }}{\boldsymbol{,}}{\boldsymbol{\beta }}}_{{ij}}^{{\prime} }]$ is the vector of trait-site combination fixed effects (i.e., overall mean for each trait-site combination); ${\boldsymbol{a}}=[{{\boldsymbol{a}}}_{11}^{{\prime} },{{\boldsymbol{\cdots }}{\boldsymbol{,}}{\boldsymbol{a}}}_{{ij}}^{{\prime} }]$ is the random vector of additive genetic effects distributed as ${\boldsymbol{a}}{\boldsymbol{ \sim }}{\boldsymbol{N}}\left({\boldsymbol{0}}{\boldsymbol{,}}{{\boldsymbol{\Omega }}}_{{\boldsymbol{a}}}{\boldsymbol{\bigotimes }}{\boldsymbol{A}}\right)$, where ${{\boldsymbol{\Omega }}}_{{\boldsymbol{a}}}$ is the unstructured genetic (co)variance matrix for each of combination of the three traits and the three sites with dimension 9 × 9. Finally, ${\boldsymbol{e}}=[{{\boldsymbol{e}}}_{11}^{{\prime} },{\boldsymbol{\cdots }},{{\boldsymbol{e}}}_{{ij}}^{{\prime} }]$ is the vector of random residuals distributed as ${\boldsymbol{e}}{\boldsymbol{ \sim }}{\boldsymbol{N}}{\boldsymbol{(}}{\boldsymbol{0}}{\boldsymbol{,}}{\boldsymbol{R}}{\boldsymbol{\bigotimes }}{\boldsymbol{I}}{\boldsymbol{)}}$ where ${\boldsymbol{R}}$ is the residual (co)variance matrix with dimension 9 × 9 between traits within sites; the residual (co)variance between traits across sites is assumed to be zero for the three sites, given that the sites were assessed separately. The matrices ${{\boldsymbol{X}}}_{{ij}}$ and ${{\boldsymbol{Z}}}_{{a}_{{ij}}}$ related the adjusted-phenotypes to the means of the trait-site combinations in ${{\boldsymbol{\beta }}}_{{ij}}^{{\prime} }$ and the genetic effects in ${{\boldsymbol{a}}}_{{ij}}^{{\prime} }$. In order to fit the GBLUP-A model, the pedigree-based relationship ***A***-matrix of the multi-site model [1] and the multi-trait multi-site model [2] was replaced by the ***G***-matrix (VanRaden 2008):

$${\boldsymbol{G}}{\boldsymbol{=}}\frac{{\boldsymbol{WW}}{{ ^\prime{} }}}{2\sum _{k}{p}_{k}(1-{p}_{k})}$$

where, ${\boldsymbol{W}}$ is the ***centered*** matrix of SNP covariates, and ${p}_{k}$ is the current (or observed) allele frequency of the genotyped trees for marker *k*.

Finally, the extended multi-site GBLUP model that included the dominance $\left({\boldsymbol{d}}=[{{\boldsymbol{d}}}_{1}^{{\prime} },{{{\boldsymbol{d}}}_{2}^{{\prime} }{\boldsymbol{,}}{\boldsymbol{d}}}_{3}^{{\prime} }]\right)$ and the additive by dominance epistatic $\left({\boldsymbol{p}}=[{{\boldsymbol{p}}}_{1}^{{\prime} },{{{\boldsymbol{p}}}_{2}^{{\prime} }{\boldsymbol{,}}{\boldsymbol{p}}}_{3}^{{\prime} }]\right)$ genetic effects (GBLUP-ADE) were fitted for each trait. These dominance and epistatic genetic effects are distributed as ${\boldsymbol{d}}{\boldsymbol{ \sim }}{\boldsymbol{N}}\left({\boldsymbol{0}}{\boldsymbol{,}}{{\boldsymbol{\Sigma }}}_{{\boldsymbol{d}}}{\boldsymbol{\bigotimes }}{\boldsymbol{D}}\right)$ and ${\boldsymbol{p}}{\boldsymbol{ \sim }}{\boldsymbol{N}}\left({\boldsymbol{0}}{\boldsymbol{,}}{{\boldsymbol{\Sigma }}}_{{\boldsymbol{p}}}{\boldsymbol{\bigotimes }}{\boldsymbol{E}}\right)$, respectively, where ${{\boldsymbol{\Sigma }}}_{{\boldsymbol{d}}}$ and ${{\boldsymbol{\Sigma }}}_{{\boldsymbol{p}}}$ are the (co)variance matrices of dominance and additive by dominance (epistatic) genetic effects, and ${\boldsymbol{D}}$ and ${\boldsymbol{E}}$ are the average dominance and additive by dominance relationship matrices, respectively. Following Gamal El-Dien et al. (2016), the average dominance relationship matrix ${\boldsymbol{D}}$ was computed using the R function Gmatrix from the R-package (http://www.r-project.org) “AGHmatrix” (Amadeu et al. 2016) using Vitezica et al. (2013)’s method. The average relationship matrix for the additive by dominance epistatic effects was computed using the Hadamard product of the additive and dominance average relationship matrices (Muñoz et al. 2014).

### Pedigree network

We generated network visualization for the ***A***- and ***G***-matrices (Rincent et al. 2012) in which two individuals are either linked, when their estimated pairwise relationship coefficient is larger than 0.05 and 0.10 or else unlinked. The observed difference in networks topology between the 0.05 and 0.10 thresholds highlights the role of ancestral pedigree. We used the R-package (http://www.r-project.org) “network” (Butts 2008) to generate the network representation.

### Heritability estimates

Average across-site pedigree- and genomic-based narrow-sense individual heritability values for each trait *i*^*th*^, ${h}_{i}^{2}$, were estimated as: ${\hat{h}}_{i}^{2}={\hat{\sigma }}_{{a}_{i}}^{2}/({{\hat{\sigma }}_{{a}_{i}}^{2}+\hat{\sigma }}_{{e}_{i}}^{2})$, where ${\hat{\sigma }}_{{a}_{i}}^{2}$ is the estimated genetic variance for the *i*^*th*^ trait, and ${\hat{\sigma }}_{{e}_{i}}^{2}$ is the estimated residual variance for the *i*^*th*^ trait from the multi-site model [1] and multi-trait multi-site model [2]. For the GBLUP-ADE models, the denominator of the above equation also included the estimated variance of dominance and epistatic effects.

Variance components and their respective heritability estimates for the ABLUP and GBLUP-based SNP selection methods were estimated in R (www.r-project.org), with the function remlf90 from the ‘breedR’ package (Muñoz and Sanchez 2020) using the Expectation-Maximization (EM) algorithm followed by one round of an Average Information (AI) algorithm to compute the standard deviations (Chateigner et al. 2020) for the variance components and heritability estimates. The remlf90 function in R-package ‘breedR’ is based on REMLF90 (for the EM algorithm) and AIREMLF90 (for the AI algorithm) of the BLUPF90 family (Misztal et al. 2018). The program postGSF90 from the BLUPF90 family (Aguilar et al. 2019) was also used to estimate SNP effects.

### SNP selection protocol

We examined the impact of SNP-marker number on the estimated genomic-based narrow-sense heritability and the theoretical accuracies of breeding values (equation [3] below) using the multi-site model [1]. To that end, subsets of 1096, 2192, 3288, 4384, 5479, 6575, 7671, and 8767 SNPs (increments of ≈1000) were randomly selected from all available SNP data and were used to build the corresponding genomic additive relationship matrices. In addition, the impact of SNP-marker type and number on heritability and theoretical accuracy was studied using additional two SNP selection strategies; namely, (1) based on their minor allele frequency (MAF) with increasing order from the rarest to the most common, and (2) based on their GWAS absolute value effect ranked from the largest to the smallest and averaged across sites using a single-step multi-site genome-wide association analysis (ssGWAS) (Aguilar et al. 2019; Uffelmann et al. 2021), using the same SNP increments as above (i.e., ≈1000).

To unravel the overlap of SNPs across traits selected by their effects, a Venn Diagram was built using subsets of 3288 SNPs each. Thereafter, the total of 5628 SNPs (Supplementary Information, Fig. S1) was used to generate a combined ***G***-matrix associated with the three studied traits.

### Genetic parameters theoretical accuracy

Estimates of the theoretical accuracy (TA) of the additive genetic variance were used to compare different genomic- and pedigree-based analytical methods. Therefore, the TA of tree *i* was calculated across all traits and ABLUP and GBLUP models using the following equation:

3 $${{\rm{TA}}}_{i}=\sqrt{1-\frac{{{\rm{PEV}}}_{i}}{{\hat{\sigma }}_{a}^{2}}}$$

where PEV_*i*_ (prediction error variance) that corresponds to individual *i* is obtained from the diagonal entry of the inverse of the coefficient matrix derived from the mixed model equations (MME).

### Optimum contribution selection (OCS)

To select the top 30 individuals for the product development phase (i.e., seed orchard establishment), we implemented OCS to optimize the trade-off between the genetic gain (Δ*G*) and the degree of co-ancestry (Δ*F*) build-up. We conducted two optimizations with Δ*F* ≤ 0.25 and 0.125. Additionally, the observed negative correlation between both height and diameter and wood density required the implementation of an additional constraint in which we maintained the wood density of the selected individuals to be similar to the base population (i.e., no wood density loss). The optimizations were conducted using Gurobi 10.0 (https://www.gurobi.com) (Gurobi Optimization 2023).

### Availability of R code

Software code(s) used in the present study are posted in the Dryad Digital Repository, https://datadryad.org/stash/dataset/doi:10.5061/dryad.7h44j101d.

## Results

One of the most critical aspects of using the ***G***-matrix in quantitative genomic analyses is the selection of a subset of SNPs that provide the most informative pairwise kinship information among individuals (Bernardo 2014). To address this, we implemented three SNP sampling approaches to construct the ***G***-matrix and compared the results to those obtained using the entire SNP sample (≈9 K). These SNP sampling methods consecutively selected subsets of ≈1000 SNPs based on: (i) random sampling (with 10 replications), (ii) rare allele frequency (ranking SNPs from rarest to most common), and (iii) GWAS-based selection (ranking SNPs based on their GWAS absolute value regardless of their statistical significance threshold to circumvent the statistical threshold limitations of GWAS and leverage potential LD between SNPs and causal genes). In this context, GWAS-SNPs were incorporated in a sequential manner without imposing specific lower inclusion criteria, aside from considering their absolute effect. To evaluate the proposed framework, we compared pedigree- vs. genomic-based approaches in terms of pedigree network and theoretical breeding value accuracy and heritability estimates.

### Pedigree networks

**Fig. 2:**
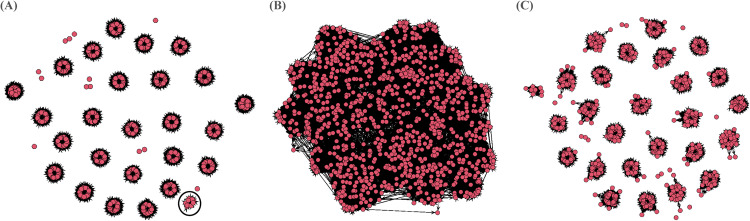
Pedigree networks. Pedigree (**A**)- and genomic-based networks with a minimal genomic pairwise kinship threshold set at 0.05 (**B**) and 0.10 (**C**) for 1101 Interior spruce individuals with the 5628 markers used in the multi-trait multi-site GWAS analysis. The black circle in (**A**) identifies the added 9-member OP family.

When genomic pairwise kinship threshold of 0.05 is considered, the comparison yielded astonishing results insofar as the pedigree-based approach produced 26 (the original 25 as well as the added small 9-member family (see pedigree verification above)) independent entities representing the tested HS families, along with 13 mislabelled individuals, while the genomic-based showed complete connectedness among all individuals, including the mislabelled, and generated a complete pairwise kinship matrix leveraging both contemporary and ancestral pedigrees (Fig. 2A, B). Naturally, the degree of connectedness among individuals is dependent on the set minimum kinship level used. It is interesting to note that the network topology between the 0.05 and 0.10 thresholds resulted in a different degree of connectedness among individuals with lower connectedness for the 0.10 threshold (Fig. 2C); however, the ***G***-matrix used in our quantitative genomics analyses considered all possible pairwise relationships irrespective of the threshold. To leverage both contemporary and ancestral pedigree, we did not impose any pairwise kinship lower limit and used the complete set of 8767 SNPs or subsets (3288 for single trait and 5628 for multi-traits) in our analyses. As indicated above, mislabelling is common in traditional tree breeding, leading to erroneous genetic parameters, ranking, and gain estimates (Muñoz et al. 2014).

### Single-trait multi-site analyses

**Fig. 3:**
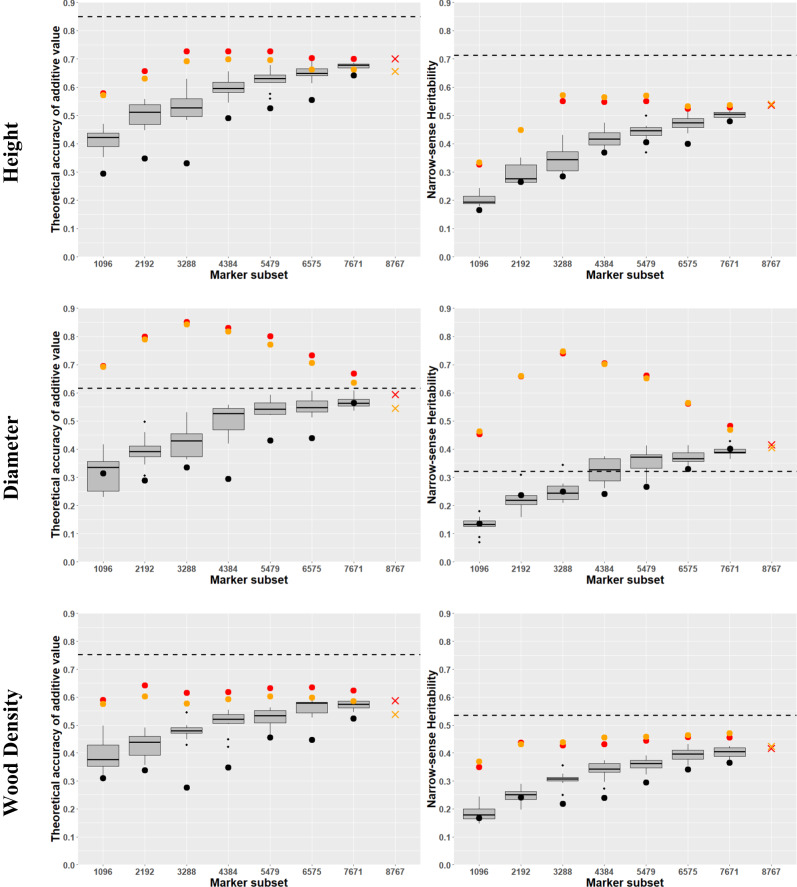
Theoretical accuracy and narrow*-*sense heritability of breeding values. Estimates are shown with SNP-marker increments ranging from 1096 to 8767. Boxplots illustrate results for 10 replications of random SNP selection; black circles indicate SNPs ranked by minor allele frequency from rarest to most common; red and orange circles represent SNPs selected based on their GWAS absolute values ranked from largest to smallest effects for additive and additive, dominance, and epistatic genomic-based GBLUP models, respectively; dashed black line indicates the theoretical accuracy and narrow-sense heritability estimates for the average breeding values from the conventional pedigree-based ABLUP model; red and orange “x” marks show theoretical accuracy and narrow-sense heritability estimates for the breeding values using the additive and additive, dominance, and epistatic genomic-based GBLUP models with the full set of 8767 SNPs.

As expected, the theoretical accuracies of ABLUP derived additive genetic variances were higher than their GBLUP counterparts. However, the DBH-ABLUP estimates overlapped with those from the different SNP sampling methods and sample sizes (Fig. 3). Generally, the GWAS additive genetic variance theoretical accuracies were better compared to random and rare allele sampling. Plateaued accuracy was observed between 3000 and 4000 SNPs, indicating that selecting GWAS-informative SNPs added significant value to the resultant ***G***-matrix (Fig. 3). Furthermore, the difference in theoretical accuracy between the two GBLUP models (additive (GBLUP-A) vs. additive, dominance and epistatic (GBLUP-ADE)) was negligible, suggesting minimal dominance and epistatic variance of these traits (Fig. 3). Notably, the additive genetic variance theoretical accuracy of the complete SNP set (8767) was lower than that obtained from the reduced GWAS-SNP set, suggesting that adding more SNPs after reaching the optimal number is detrimental to the resulting ***G***-matrix (Fig. 3).

### Heritability estimates

The heritability estimates exhibited a trend similar to the additive genetic variance theoretical accuracies. Importantly, the GWAS-SNP sampling yielded higher estimates compared to random and rare allele sampling (Fig. 3). Overall, ABLUP produced higher heritability estimates than GBLUP, primarily due to the ABLUP inflated additive genetic variance (Beaulieu et al. 2022). Our results showed no significant differences between the GBLUP-A and GBLUP-ADE models, further supporting minimal dominance and epistatic genetic variances (Table 1).

**Table 1:** Single*-*trait multi*-*site and multi*-*trait multi*-*site average heritability.

Single*-*trait multi*-*site			
Trait	ABLUP	GBLUP*-*A	GBLUP*-*ADE
HT	0.71 (0.06)	0.54 (0.09)	0.54 (0.09)
DBH	0.32 (0.16)	0.42 (0.21)	0.40 (0.21)
WD	0.54 (0.20)	0.42 (0.17)	0.42 (0.17)

Multi*-*trait multi*-*site			
Trait	ABLUP	GBLUP*-*ALL	GBLUP*-*GWAS
HT	0.69 (0.02)	0.52 (0.09)	0.62 (0.09)
DBH	0.34 (0.01)	0.44 (0.21)	0.66 (0.21)
WD	0.52 (0.01)	0.43 (0.17)	0.52 (0.17)

Heritability estimates (standard error) for heigh (HT), diameter (DBH), and wood density (WD) for average numerator relationship (ABLUP), additive genomic-based (GBLUP-A), and additive, dominance and epistatic genomic-based (GBLUP-ADE) relationships (GBLUP-ALL: full 8767 SNPs and GBLUP-GWAS: 5628 SNPs selected by GWAS absolute effects).

### Multi-trait multi-site analysis

Breeding programs select for multiple traits while considering the “correlated response” among traits. Our data revealed negative genetic correlations between wood density and both height (−0.53) and diameter (−0.72) (Supplemental Fig. S2). Furthermore, the GWAS-SNP single-trait multi-site analyses indicated that 3000 to 4000 was the optimal number of SNPs needed for obtaining reliable genetic parameters (Fig. 3) and a SNP overlap existed among the studied traits (Supplemental Fig. S1). Thus, we constructed a new ***G***-matrix from each trait’s top 3288, resulting in a total of 5628 SNPs that were subsequently used for further analyses. Overall, the ABLUP multi-trait multi-site heritability estimates were comparable to those of the single-trait multi-site, and the GWAS-SNP approach yielded better estimates than the entire SNP sample (Table 1). The superiority of the GBLUP-GWAS heritability estimates can be attributed to the refinement of residual variances, which removed the influence of dominance and epistatic genetic variances as well as their interactions with environments, from the “error” term. This refinement reduced their respective denominators and produced higher estimates and thus enhanced the expected genetic gain as compared to those from the ABLUP models (Table 1). Ignoring the inflated additive genetic variance and hereditability estimates produced and the time saving associated with use of OP families, we compared the percentage of expected genetic gain between the ABLUP and GBLUP models after selecting the top 30 individuals for improved seed production (El-Kassaby 1995). The results showed greater gains from the GBLUP compared to ABLUP for two traits (diameter: 8.18 vs. 4.35% and wood density: 14.91 vs. 10.77%), while height gain remained consistent (10.77 vs. 10.88%), supporting our proposed framework.

### Optimum contribution selection (OCS)

**Fig. 4:**
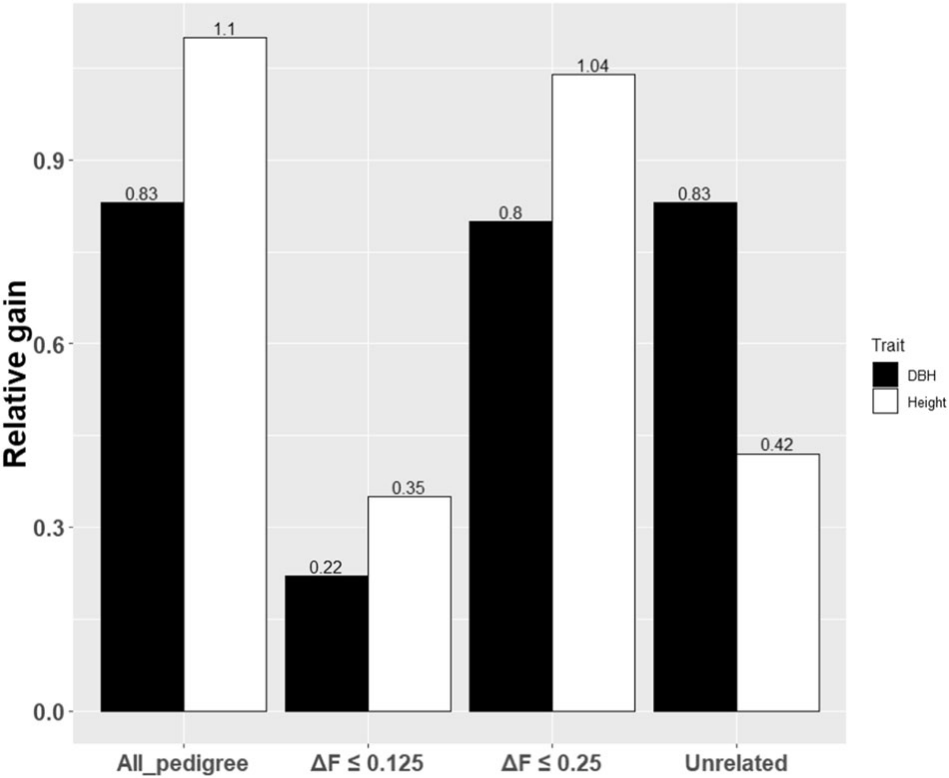
Comparison of the relative gains from optimum contribution selection between ABLUP and GBLUP*-*GWAS. ABLUP is represented by ‘All_pedigree’, including all individuals in the pedigree and reflecting no co-ancestry constraint, and by ‘Unrelated’ having only unrelated individuals. Results of GBLUB-GWAS show the gains under Δ*F* ≤ 0.25 (Δ*F* ≤ 0.25) and Δ*F* ≤ 0.125 (Δ*F* ≤ 0.125) for diameter (DBH) and height.

The translational component of breeding programs is the production of improved seed, which in forestry is achieved through establishing seed orchards comprising the highest genetic worth individuals. We implemented OCS (Woolliams et al. 2015) to select the top 30 individuals and optimized genetic gain (Δ*G*)/co-ancestry (Δ*F*) trade-off, with an additional constraint to prevent wood density loss. Regardless of the analytical method used (ABLUP or GBLUP), the highest relative gains of 0.83 (diameter) and 1.10 (height) were achieved under no co-ancestry constrain (i.e., individuals were selected irrespective of their family affiliation) (Fig. 4). Compared to the unconstrained co-ancestry approach, the unrelated ABLUP resulted in diameter (0.83: 100%) and height (0.42: 35%) gains with only seven individuals, an insufficient number to maintain broad genetic diversity (Fig. 4). Under Δ*F* ≤ 0.25 constraint, the GBLUP-GWAS produced gains comparable to those under no constraint, yielding diameter (0.80: 96%) and height (1.04: 92%) gains with 30 carefully selected individuals. Under Δ*F* ≤ 0.125 constraint, reduced gains in diameter (0.22: 27%) and height (0.35: 32%) were obtained with only 24 individuals (Fig. 4). The superiority of OCS was clearly demonstrated in successfully capturing a significant portion of the available gains without compromising co-ancestry, specifically for Δ*F* ≤ 0.25. It should be noted that the implementation of OCS was critical in avoiding the pitfalls of exclusive genomic selection (GBLUP-GWAS), which tends to select related individuals, resulting in the accumulation of co-ancestry and depletion of genetic variation (Sonesson et al. 2012).

## Discussion

During the 1950s, large-scale selective tree breeding programs were initiated worldwide (White et al. 2007). Despite variation in geography, species, and breeding strategies, most programs followed the recurrent selection, with some reaching their fourth generations (Jing et al. 2023), yet limited pragmatic changes have been implemented (Cotterill 1986). These breeding programs involve thousands of parents and their crosses, and the resulting offspring are evaluated over multiple test sites located within expansive geographic areas known as breeding zones (White et al. 2007). These test sites exhibit considerable heterogeneity, requiring innovative experimental designs (Libby and Cockerham 1980) and statistical analyses that account for spatial and competition effects (Cappa et al. 2017) to separate genetic and non-genetic effects. Historically, these breeding zones were considered “static”, and the focus was on identifying individuals for future breeding or inclusion in seed orchard populations. However, due to climate change, these breeding zones are now in constant state of flux (Cortés et al. 2020). The existing traditional test sites, with their confounding factors and evolving environmental heterogeneity, are inadequate for addressing crucial climate change questions, such as identifying genotypes that are resilient to abiotic and biotic stresses (Surówka et al. 2020). Additionally, conventional breeding methods necessitate using structured pedigree (White et al. 2007), which is time-consuming and impractical and can be overcome using the proposed framework.

The effectiveness of the proposed selective breeding framework can be illustrated within the context of the breeder equation (ΔG = *i r σ*_*a*_ / *L*), where ΔG, *i*, *r*, *σ*_*a*_, and *L* represent the genetic gain, selection intensity, accuracy of selection, additive genetic variance estimate, and breeding cycle length, respectively (Lush 1937). When considering equal *i* for ABLUP and GBLUP, the GBLUP yields higher estimates for *r* and *σ*_*a*_. Furthermore, if OP families are utilized, the breeding cycle length (*L*) would be significantly shortened for bypassing the structured pedigree requirement, confirming the superiority of our approach. Additionally, traditional tree breeding programs required extensive pedigree control to mitigate the adverse effects of inbreeding depression (Williams and Savolainen 1996). Our results showed pedigree errors (***A***-matrix); thus, it is suggested that the ***G***-matrix information should be employed for effective pedigree and genetic diversity management (El-Kassaby et al. 2019).

Despite the considerable size of the spruce genome (20 Gb; Birol et al. 2013) and the “limited” likelihood of our ≈9000 SNPs being in linkage disequilibrium (LD) with the studied traits, we have confidence in the efficacy of our method for the following reasons: (1) it is widely recognized that 3000 to 4000 SNPs are generally sufficient for resolving relatedness (Thistlethwaite et al. 2020), and our SNP count falls within this range and (2) the selected SNPs have been established to exert an effect on the studied attributes through GWAS, even if these effects are modest. Collectively, they still contribute to explaining a portion of the variance in the studied traits. Therefore, we believe that despite the relatively small number of SNPs (≈9000) used in our study, these selected SNPs effectively capture some of the variance in the traits, with the added advantage of elucidating the pedigree.

Comparing the proposed genomic-based approach with the two previously used “time-saving” testing and evaluation methods (i.e., OP testing and pedigree reconstruction (Breeding without Breeding)) can be summarized as follows: (1) while both methods capitalize on the readily available natural matings and invoke either half-sib (OP families) or full-sib (pedigree reconstruction) family structure and thus avoiding the creation of structured pedigree (i.e., the breeding phase), the OP testing produces inflated additive genetic vicariance and heritability estimates (Askew and El-Kassaby 1994) which the pedigree reconstruction considers (El-Kassaby and Lstibůrek 2009) and (2) both OP testing and pedigree reconstruction are anchored on the use of the ***A***-matrix and therefore both do not benefit from the information gained from ancestral pedigree (VanRaden 2008) and thus hidden relationships and inbreeding are not accounted for.

Lastly, it is essential to underscore that the presented framework is exceptionally well-suited for evaluating climate change-related adaptive attributes. These attributes can be conveniently assessed at a very young age owing to their notably high heritability values. Examples of such attributes include vegetative phenology (Guo et al. 2021), drought tolerance (Moran et al. 2017), frost tolerance (Gomory et al. 2010), salt tolerance (Khasa et al. 2002), and insect resistance (Klápště et al. 2022).

## Conclusions

Tree selective breeding methods have remained static since their inception, with no pragmatic changes implemented to expedite the process or to address environmental contingences. Here, we introduced an innovative approach that integrates genomic data and optimization protocols for evaluating and selecting superior individuals. The proposed framework leverages the existing OP families, eliminating the need for the traditional breeding phase and resulting in greater gains with a shorter timeline.

### Supplementary information

Supplementary Information

## Data Availability

All data sets used are available in the Dryad Digital Repository, 10.5061/dryad.7h44j101d.

## Code availability

Software code(s) used in the present study are posted in the Dryad Digital Repository, https://datadryad.org/stash/dataset/doi:10.5061/dryad.7h44j101d.
