# Supplementary material for: Efficient genomics-based ‘end-to-end’ selective tree breeding framework
Source: Heredity (Edinb). 2024 Jan 3;132(2):98–105. doi: 10.1038/s41437-023-00667-w (PMC10844606; doi:10.1038/s41437-023-00667-w)
Supplement: Supplementary file 1 — Supplementary Information [file 41437_2023_667_MOESM1_ESM.docx]

**Supplementary Information**

Fig. S1 Venn diagram showing SNP overlaps among the three studied Interior spruce traits (HT: height; DBH: diameter; WD: wood density) for the 3,288 SNPs sampled from each trait-GWAS.


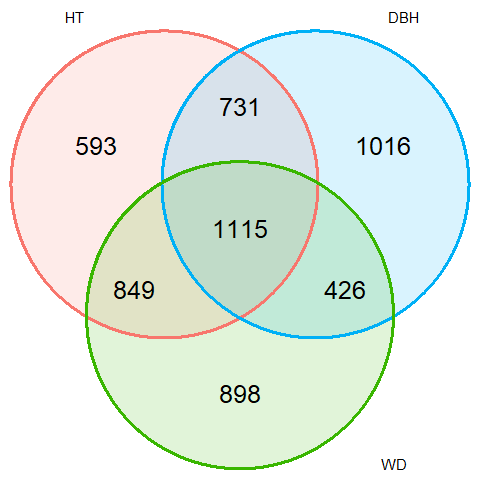


Fig. S2 Correlations between tree height (HT), diameter (DBH), and wood density (WD) for 1,101 Interior spruce individuals.

| 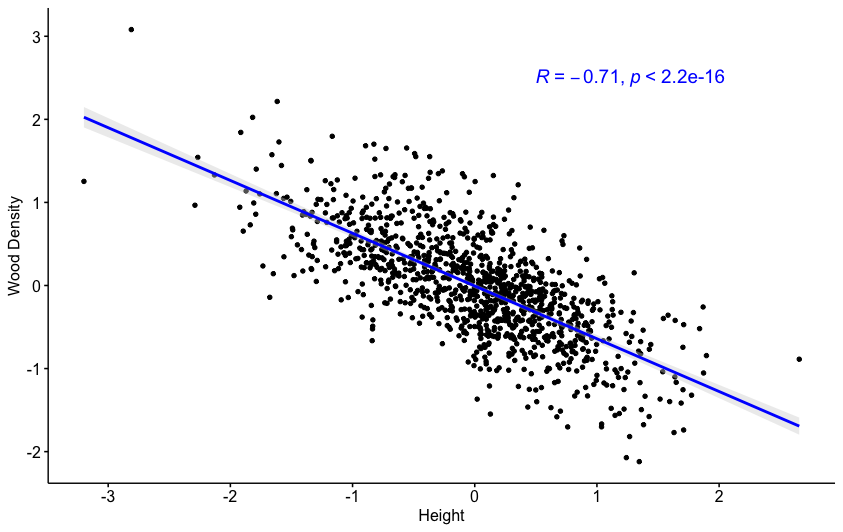 | 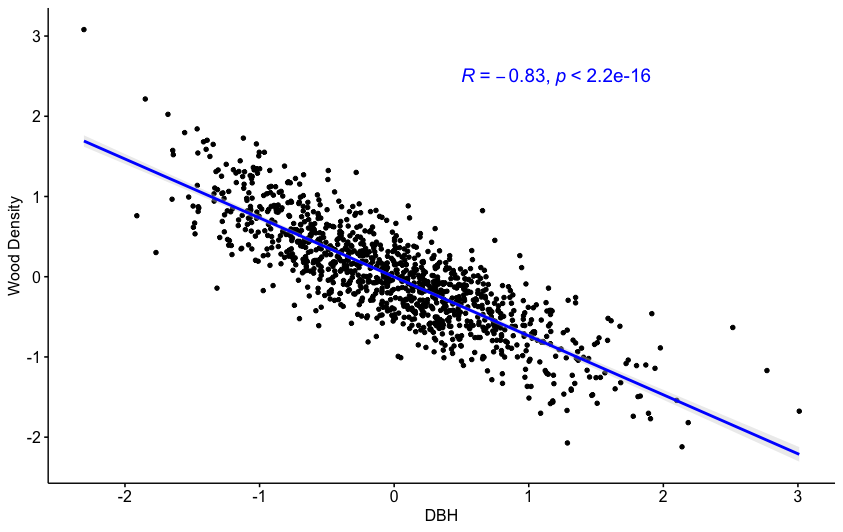 | 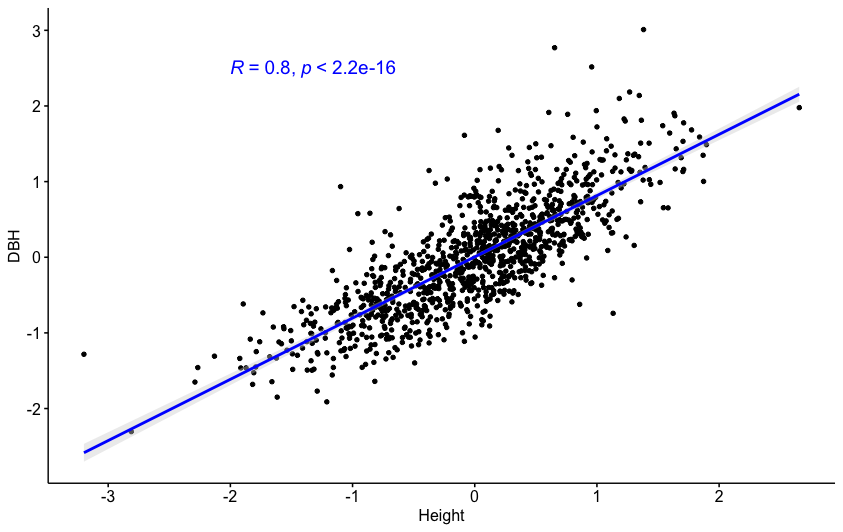 |
| --- | --- | --- |
